# Discovery of 12-mer peptides that bind to wood lignin

**DOI:** 10.1038/srep21833

**Published:** 2016-02-23

**Authors:** Asako Yamaguchi, Katsuhiro Isozaki, Masaharu Nakamura, Hikaru Takaya, Takashi Watanabe

**Affiliations:** 1 Research Institute for Sustainable Humanosphere, Kyoto University, Gokasho Uji, Kyoto 611-0011, Japan; 2 Institute for Chemical Research, Kyoto University, Gokasho Uji, Kyoto 611-0011, Japan

## Abstract

Lignin, an abundant terrestrial polymer, is the only large-volume renewable feedstock composed of an aromatic skeleton. Lignin has been used mostly as an energy source during paper production; however, recent interest in replacing fossil fuels with renewable resources has highlighted its potential value in providing aromatic chemicals. Highly selective degradation of lignin is pivotal for industrial production of paper, biofuels, chemicals, and materials. However, few studies have examined natural and synthetic molecular components recognizing the heterogeneous aromatic polymer. Here, we report the first identification of lignin-binding peptides possessing characteristic sequences using a phage display technique. The consensus sequence HFPSP was found in several lignin-binding peptides, and the outer amino acid sequence affected the binding affinity of the peptides. Substitution of phenylalanine7 with Ile in the lignin-binding peptide C416 (HFPSPIFQRHSH) decreased the affinity of the peptide for softwood lignin without changing its affinity for hardwood lignin, indicating that C416 recognised structural differences between the lignins. Circular dichroism spectroscopy demonstrated that this peptide adopted a highly flexible random coil structure, allowing key residues to be appropriately arranged in relation to the binding site in lignin. These results provide a useful platform for designing synthetic and biological catalysts selectively bind to lignin.

The depletion of fossil resources and the increase in atmospheric carbon dioxide concentrations have motivated the establishment of biorefinery processes that utilise lignocellulosic plant biomass as fuels and chemicals. The main components of lignocellulosic biomass are carbohydrate polymers in the form of cellulose, hemicellulose, and the aromatic polymer lignin. Lignin is a highly complex aromatic heteropolymer composed of 4-hydroxycinnamyl alcohol (H), coniferyl alcohol (G), and sinapyl alcohol (S), interlinked by ether and carbon-carbon bonds. Lignin plays a central role in providing physical, biological, and chemical stability to plant cell walls by coating polysaccharides, cellulose, and hemicelluloses within the cell wall^1^,^2^,^3^. Because of the crucial functions of lignin in maintaining the cell wall architecture, lignin degradation has emerged as a key technology for lignocellulosic biorefineries^1^,^2^. Disintegration of the lignin network and subsequent hydrolysis of cell wall polysaccharides in conjunction with the conversion of lignin into high value-added products would greatly improve the economics of the overall biomass conversion process. However, efficient degradation of lignin in plant cell walls remains a challenge due to the recalcitrance of their chemically and physically stable aromatic-rich backbone and the limited accessibility of enzymes and synthetic catalysts to the molecules incorporated into cell wall networks^1^,^2^,^3^,^4^.

Various lignin degradation methods using chemical catalysts have been developed^1^,^3^,^4^,^5^,^6^. Therefore, identification of lignin-binding peptides and incorporation of such peptides into synthetic catalysts would be expected to increase the selective recognition of catalysts to lignin, thereby promoting lignin degradation through increased accessibility of the catalysts to the lignin in plant cell walls^7^.

In the biodegradation of structurally heterogeneous lignin by wood-rotting basidiomycetes, lignin-degrading enzymes such as laccases and peroxidases play a critical role^8^,^9^,^10^,^11^,^12^,^13^,^14^,^15^,^16^. Lignin-degrading enzymes extract one electron directly from polymeric lignin or through mediators. In the former case, direct contact of the enzymes with lignin is necessary. In the latter case, diffusible or enzyme-bound mediators can transfer electrons from lignin to the enzymes; even for diffusible mediator systems, enzymes should be located at a site close to the vicinity of the substrate because the life span of highly reactive mediator radicals is very short^17^, and the active radicals readily react with various organic molecules that they encounter. Reactions of the radicals with cell wall polysaccharides^18^ decrease the selectivity for lignin-degrading reactions, as suggested by enhanced radical-mediated degradation of cellulose by adsorption of redox-active transition metals on cellulose^19^. Thus, the binding of lignin-degrading enzymes to lignin would be important both through direct contact and mediator systems; however, no specific amino acid sequences have been characterized as the binding motif in ligninolytic enzymes. The identification of such lignin-binding peptides will provide a basic understanding of lignin-adsorbing mechanisms by specific amino acid sequences that may be useful to design new types of enzymes and catalysts with increased or decreased affinity for lignin. Moreover, this approach would also allow the catalysis of lignin polymerisation in plant cell walls to be controlled using laccases and peroxidases, which have distinct affinities for growing lignin molecules in plants.

Phage display techniques are powerful tools for identifying new proteins and/or peptides that specifically bind to various target molecules^20^. Phage peptide libraries are comprised of random DNA sequences, which encode various peptides, fused on the phage gene. Recombinant peptides displayed on the surface of bacteriophage have the ability to recognize target molecules. After an *in vitro* selection process based on binding affinity, the selected peptide are characterized by DNA sequencing. Through this technology, the detection of ligands as specific targets is accomplished without an animal or human immunization system. Recently, this technology has been used for immunological and biological studies and has been applied to inorganic materials^21^,^22^ and artificial material surfaces^23^. Peptides having affinities for a variety of proteins, including enzymes, cell-surface receptors, and antibodies, have been available as bioactive molecules, antibiotic molecules, and novel enzyme substrates^24^,^25^,^26^. Peptides that recognize metals or synthetic polymers with simple chemical structures have already been isolated and are available for the development of multifunctional hybrid materials and immobilization technology^20^,^21^,^22^,^23^. However, no previous reports have described the targeting of phage display experiments to isolated lignin.

Here, we report the first lignin-binding peptides identified using a phage display system.

## Results

### Selection of peptides having affinity for lignin

**Figure 1:**
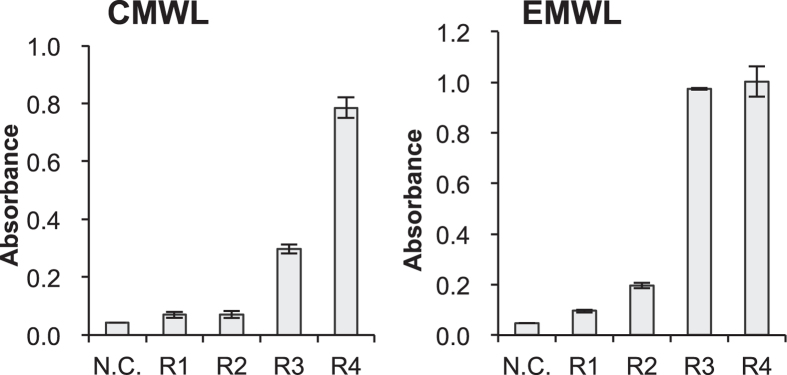
Lignin-binding strengths of eluted phages after each biopanning round (R1–R4), measured for CMWL and EMWL. After each round, the eluted pools were amplified and diluted to a phage concentration of 10^9 ^pfu/mL. Absorbance measured without adding any phage was used as the negative control (N.C.).

We applied phage display to screen for peptides that bind to lignin with high affinity. Milled wood lignins (MWLs) derived from a softwood, Japanese cedar (CMWLs), and MWLs derived from a hardwood, *Eucalyptus globulus* (EMWLs), were used as biopanning targets. Softwood lignin is composed mostly of guaiacyl phenylpropanoid (G) units, whereas hardwood lignin consists principally of G and syringyl phenylpropanoid (S) units^1^,^2^. A phage library displaying random dodecapeptides was incubated with the softwood and hardwood lignins, and the bound phages were isolated after four rounds of biopanning. Phage yields from each round were used to indicate the enrichment of phages in each round (Table 1). The affinity of the bound phages for lignin was analysed using enzyme-linked immunosorbent assay (ELISA) plates coated with CMWLs or EMWLs. After each round of biopanning, the bound phages were recovered using an elution buffer, and the results of phage ELISA performed using these eluates demonstrated that the lignin-binding peptides increased as the biopanning rounds increased (Fig. 1).

### Analysis of peptide amino acid sequences

Phage clones were randomly isolated from the third and fourth rounds of biopanning. Sequencing the DNA of these clones allowed for determination of the amino acid sequences of the displayed peptides. From the 70 selected clones, 55 distinct peptide sequences were determined. The 55 clones included 26 and 29 peptides that bound to CMWLs and EMWLs, respectively (Table 2). The peptides C416 (HFPSPIFQRHSH) and E405 (VQHNTKYSVVIR) were found at the highest frequencies for CMWLs and EMWLs, respectively. The sequence YHPNGMNPYTKA appeared in the biopanning for both CMWL (C401) and EMWL (E407).

In addition, these peptide sequences were analysed by searching the UniProtKB/Swiss-Prot database using BLAST. The peptide C416 shared 89% identity with deuterolysin (Seq ID: EUC58573.1), a microbial zinc-containing metalloprotease. Additionally, the peptide C401 shared 89% identity with uroporphyrin-III C-methyltransferase (Seq ID: WP_008750060), which is involved in heme synthesis. The peptide E405 shared 73% identity with phosphoribosylaminoimidazole carboxylase (Seq ID: WP_014940321), which is involved in purine biosynthesis. Although the functions of the individual enzymes were not related to lignin binding, the structural similarities suggested that the peptide sequence could be incorporated into protein structures.

The frequencies of the amino acid residues present in the peptides selected using biopanning are tabulated in Table 3. Our results showed that His was present at a high frequency for both hardwood (10.65%) and softwood (10.25%) lignin. Pro appeared at a high frequency for softwood lignin (13.24%), whereas Asn and Val appeared at high frequencies for hardwood lignin (8.10% and 8.56%). In contrast, Cys was not a component of any peptide selected for EMWLs, and its frequency in the case of CMWLs was negligible.

The isoelectric points (p*I*s) of lignin-binding peptides ranged from 4.0 to 12.0 (as shown in Table 2). The p*I* values of all the lignin-binding peptides that appeared at high frequency, such as C416, C401, and E405, were basic (8.5–10.0), and only two acidic peptides (p*I* < 5.0) were found among the 55 isolated peptides. In nonproductive binding of cellulases to lignin, positively charged cellulases with high p*I*s are much more favourably adsorbed on lignin than neutral and negatively charged proteins^27^. Therefore, the high frequency of selected peptides with high p*I*s was consistent with the adsorptive properties of cellulases with different p*I*s.

### Binding assay by phage ELISA

**Figure 2:**
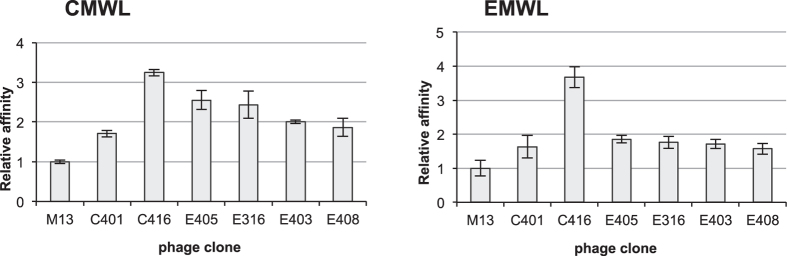
Lignin-binding affinity of phage clones analysed using phage ELISA. Relative affinities for CMWL and EMWL were determined by means of phage ELISA and are compared to the affinity measured for wild-type M13 phage. The phage solution was used at a concentration of 10^9 ^pfu/mL. Data shown are averages ± standard deviations from three experiments.

The peptides that appeared at a high frequency, i.e., C401, C416, E316, E403, E405, and E408, were selected, and their binding affinities for lignin were measured using phage ELISAs (Fig. 2). All of the selected phage clones showed higher MWL-binding affinity than did the wild-type M13 clone. Among the phage clones analysed, C416 exhibited the highest affinity both for CMWLs and EMWLs. Although the phage pools were obtained using either CMWLs or EMWLs, the selected phage clones bound to both CMWLs and EMWLs.

### Binding affinities of the synthesized peptides

**Figure 3:**
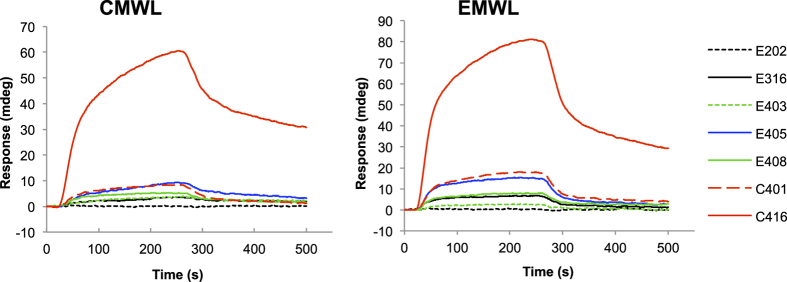
SPR sensorgrams obtained for the binding of synthetic peptides with lignin. The lignins used were CMWL and EMWL. E316 (black), E403 (green dotted line), E405 (blue), E408 (green), C401 (red dashed line), and C416 (red) were selected as the high-frequency-sequence peptides from the third and fourth rounds of biopanning. E202 (dotted line) was used as a negative control. The peptide solution (100 μM) was injected over a lignin-immobilised sensor chip; the association (15–255 s) and dissociation (255–500 s) phases are shown.

The peptides C401, C416, E202, E316, E403, E405, and E408 were chemically synthesised as free soluble peptides, and their binding affinities for lignin were determined by means of surface plasmon resonance (SPR) measurement (Fig. 3). E202 (VGNSSTRLEWDS), which was chosen randomly from the second biopanning round as a negative control, exhibited no binding affinity for lignin. The SPR angle shift measured for C416 was the highest, followed by those for C401 and E405. The peptide C416 bound to CMWLs and EMWLs with the highest affinity in both phage ELISAs (Fig. 2) and SPR assays. However, in contrast to the results obtained using phage ELISAs, in SPR measurements, the peptides E316, E403, and E408 showed small angle shifts (< 5 mdeg). E408 (KGPHFPSPHVAL), which was isolated from the biopanning performed using EMWLs, contained the HFPSP sequence that was also present in C416. However, in SPR assays, E408 bound to MWLs with considerably lower affinity than did C416. Thus, these data suggested that HFPSP was a potential lignin-binding motif but not the sole determinant of the binding affinity for lignin.

**Figure 4:**
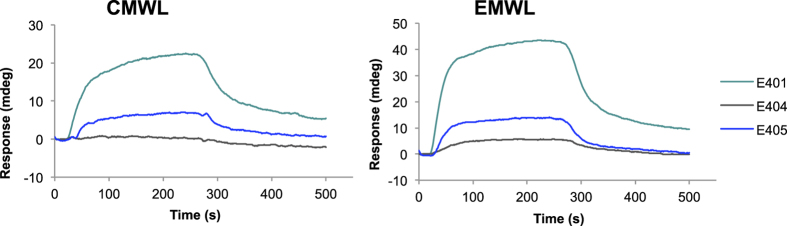
SPR sensorgrams obtained for the binding of synthetic peptides to lignin. Responses of E401 (emerald green), E404 (grey), and E405 (blue) measured using CMWL and EMWL. The peptide solution (100 μM) was injected over a lignin-immobilised sensor chip; the association (15–255 s) and dissociation (255–500 s) phases are shown.

The sequences of E401 and E404 were highly similar to that of E405 and featured only one amino acid substitution (Table 2). The affinity of E401 for the two MWLs was higher than that of E405, which indicated that the change from Thr in E405 to Arg in E401 increased the lignin-binding affinity (Fig. 4). Substitution of the hydrophobic amino acid Val in E405 by the polar amino acid Asp in E404 further decreased the binding affinity.

**Figure 5:**
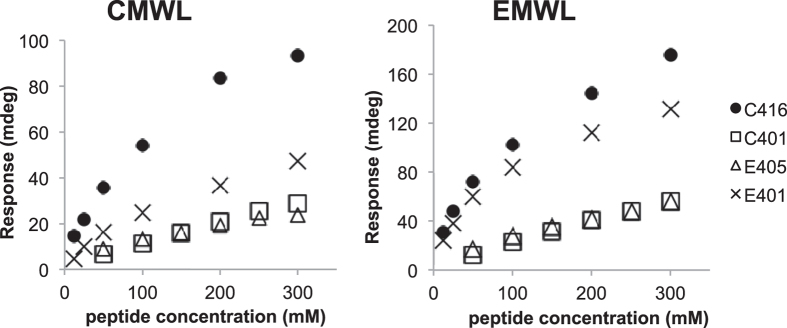
The binding responses of synthetic peptides for CMWL and EMWL, as measured using SPR. Responses after 225 s are plotted versus peptide concentration.

Next, we used SPR to analyse the binding kinetics of C401, C416, E401, and E405, and equilibrium binding experiments were performed to calculate the *K*_D_ values for these peptides using Scatchard plots^28^ of the SPR results. The smallest *K*_D_ values were measured for the binding of C416 to CMWLs and EMWLs (Fig. 5 and Table 4).

### Analysis of the amino acid residues interacting with lignin

**Figure 6:**
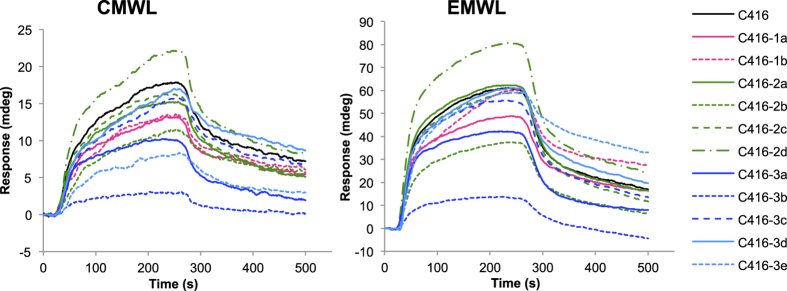
Effects of single-residue substitution on C416 binding to lignin. SPR sensorgrams obtained for C416 (black) and the following mutants: C416-1a (pink), C416-1b (pink dotted line), C416-2a (green), C416-2b (green dotted line), C416-2c (green dashed line), C416-2d (green dashed-dotted line), C416-3a (blue), C416-3b (blue dotted line), C416-3c (blue dashed line), C416-3d (sky blue line), and C416-3e (sky blue dotted line). The lignins used were CMWL and EMWL; the peptides were used at a concentration of 100 μM. Association (15–255 s) and dissociation (255–500 s) phases are shown.

To analyse how each amino acid residue in C416 contributed to the binding affinity of the peptide for lignin, single-residue-substituted mutants of C416 were synthesised (Table 5) and their binding affinities were measured using SPR (Fig. 6).

Replacement of Ser11 (C416-2d) with Ile increased the binding affinity for CMWLs and EMWLs, while replacement of Phe2 (C416-3b) with Ile markedly decreased the affinity of the peptide for MWLs. Subsequently, replacement of His1 (C416-3a) and Arg9 (C416-2b) with Ile contributed to the decrease in the affinity for MWLs. Substitution of Phe7 with Ile (C416-3e) decreased the affinity of the peptide for CMWLs, but did not change the affinity of the peptide for EMWLs, indicating that the lignin-recognition mechanism differed between CMWLs and EMWLs. Substitution of Pro (C416-1a and C416-1b) with Ile slightly decreased the affinity for MWLs.

### Structural analysis of peptides

**Figure 7:**
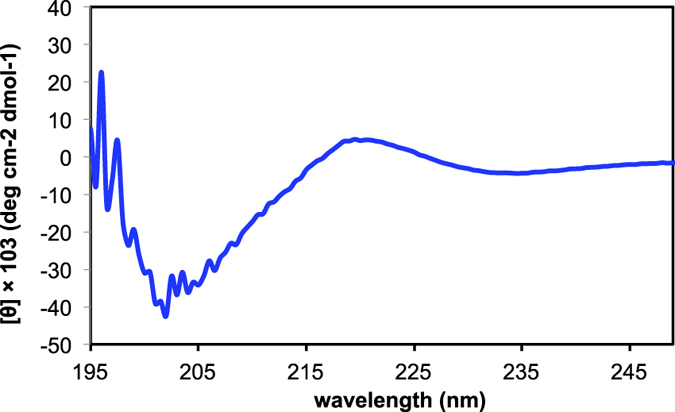
Circular dichroism spectrum of the synthesised peptide C416. The peptide concentration in water was 0.5 μM.

**Figure 8:**
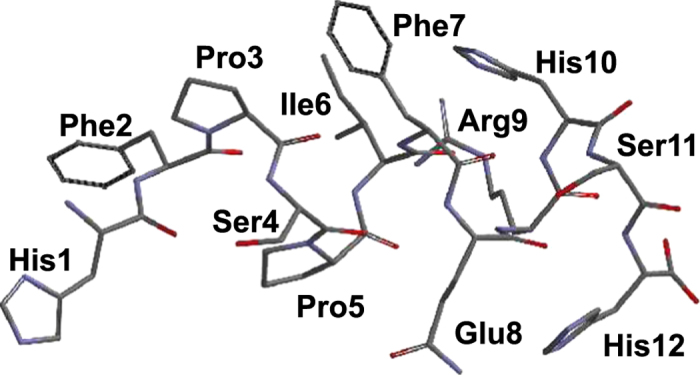
Structure of C416 generated through molecular modelling.

Peptide secondary structures are key determinants of the interaction with lignin. Therefore, we used circular dichroism (CD) spectroscopy to analyse the secondary structure of the lignin-binding peptide C416. The CD spectrum of C416 exhibited a minimum at 202 nm and a maximum at 220 nm, demonstrating that the peptide adopted a random coil structure (Fig. 7). This result suggested that C416 was highly flexible and could change its conformation dynamically in solution, thereby optimising the arrangement of amino acid residues in the peptide in relation to the binding site in lignin. The results of molecular modelling for C416 supported the formation of a helical structure (Fig. 8).

## Discussion

In this study, we successfully identified lignin-binding peptides using a phage display technique. The selected peptides were found to possess a characteristic sequence and exhibit structure-dependent high-affinity binding to the lignin isolated from softwood and hardwood.

The observed decrease in binding affinity to MWLs resulting from the substitution of Phe2 with Ile (C416-3b) suggested that π-π stacking through the phenyl group in Phe may play a major role in the adsorption of a peptide onto the lignin. A previous study suggested that the π-π stacking interaction, in addition to electrostatic and hydrogen-bonding interactions, is a major binding force in the nonproductive binding of cellulase with lignin^27^,^29^,^30^,^31^,^32^. As shown in Table 3, the enrichment of His residues in the isolated peptide pool was apparent for both CMWLs and EMWLs. Depending on the charge of its imidazole ring, His can play multiple roles in binding to organic compounds through cation-π stacking interactions, π-π stacking interactions, hydrogen-π interactions, and hydrogen bonding^33^. Given the basic p*I*s of these peptides (Table 2), cation-π stacking interactions through the guanidinium of Arg may also participate in lignin binding.

Substitution of Phe7 with Ile (C416-3e) decreased the affinity of the peptide for softwood lignin. However, the affinity for hardwood lignin did not change, indicating that the lignin-recognition mechanism differed between the G and G/S lignins. Phe and His are major components that interact with phenolic compounds in the substrate-binding sites of peroxidase and laccase^14^,^15^,^34^,^35^. Substitution of Pro (C416-1a and C416-1b) slightly decreased the lignin-binding affinity. Pro can induce a turn/kink in the peptide structure^36^. The presence of Pro probably facilitates the reduction in the structural flexibility of the dodecapeptide, thus providing a constraint configuration for ligand binding. Replacement of Ser11 (C416-2d) with Ile increased the affinity for MWLs, suggesting that the formation of hydrogen bonding through the hydroxyl group of Ser with lignin or the other amino acid residues may play an important role in the lignin-binding affinity.

CD spectroscopy suggested that this peptide adopted a highly flexible random coil structure that allowed key residues to be appropriately arranged in relation to the binding site in lignin. To the best of our knowledge, this is the first report describing the identification of lignin-binding peptides. Our study may provide the basis for the design of synthetic and natural catalysts to improve the selectivity for lignin.

Unlike polysaccharide hydrolases containing carbohydrate-binding modules^37^, ligninolytic enzymes do not contain lignin-binding modules. The lignin-binding amino acid sequences are key components controlling the binding affinity of lignin-related enzymes, which can promote the selectivity of ligninolysis and reduce nonproductive adsorption in the development of enzymatic digestion of biomass. Therefore, such lignin-binding peptides may be applicable as tools for the transformation of plant cell walls and lignin-degrading microorganisms for biorefineries. The analysis of lignin-(un)binding peptide sequences may also provide information for the development of polysaccharide-degrading enzymes, such as cellulases and hemicellulases, which are not susceptible to unproductive adsorption of lignin in pretreated biomass.

## Methods

### Materials

The Ph.D.^TM^-12 phage display peptide library and *Escherichia coli* ER2738, used as the host organism, were purchased from New England BioLabs Inc. (Ipswich, MA, USA). All laboratory reagents were from Sigma-Aldrich (St. Louis, MO, USA) and Wako Pure Chemical Ltd. (Osaka, Japan).

### Preparation of MWLs

MWLs were extracted from finely ball-milled biomass of softwood Japanese cedar (CMWLs) and hardwood *Eucalyptus globulus* (EMWLs) using aqueous dioxane as previously described^38^,^39^.

### Phage display library

Phage peptide libraries are composed of random DNA sequences that encode various peptides, which can recognise a target fused on the bacteriophages. The Ph.D.^TM^-12 phage display library of a random dodecapeptide (New England BioLabs, Inc.) was used to screen against lignin. The peptide library was displayed, with a GGGS spacer, on minor coat protein III of M13 phage. To determine the phage titres, various dilutions of the phage clones together with top agar were spread on LB/IPTG/X-gal agar plates. We estimated the phage library titre to be 3.1 × 10^13^ plaque forming units (pfu)/mL, with a complexity of 10^9^ unique peptide sequences.

### Affinity selection through biopanning

Phage selection, called biopanning, was performed using the Ph.D.^TM^-12 library kit standard procedure with certain modifications. We conducted biopanning against lignin by modifying the solution-phase panning method because lignin is insoluble in aqueous solutions. The target molecule MWL was washed 10 times with phosphate-buffered saline (PBS, pH 7.0), and the pellet was collected through centrifugation (12,000 × *g*, 10 min, 4 °C). The phage library (1.5 [10^11 ^pfu]) was incubated with MWLs in 200 μL of PBS. After 1 h at room temperature (RT), the mixture was washed 10 times with PBS containing 0.05% Tween-20 (PBST) and centrifuged (12,000 × *g*, 2 min, 4 °C) each time to remove unbound phages in the supernatant. The bound phages were eluted for 10 min with 200 μL of elution buffer (0.2 M glycine-HCl, pH 2.2, 0.1% bovine serum albumin [BSA]), and the eluate was neutralised with 1 M Tris-HCl (pH 9.1). To estimate the phage number, a small portion of the eluate was saved and used to measure the titre. Eluted phages were amplified by infection of *E. coli* ER2738 cells grown in LB medium. The amplified phages were collected and purified using PEG/NaCl (20% [w/v] polyethylene glycol [PEG]-8000, 2.5 M NaCl) and then used in the next round. This process was repeated four times, with the concentration of Tween-20 being raised from 0% to 0.1% in the binding and washing steps. After each round of affinity selection, the titre plate was used for isolating individual phage clones. Phage DNA was purified using PEG/NaCl and an iodide buffer (10 mM Tris-HCl, pH 8.0, 1 mM EDTA, 4 M sodium iodide) and used as a template for DNA sequencing. After the third and fourth rounds of biopanning, phage clones were randomly selected and their amino acid sequences were determined through DNA sequencing.

### Phage ELISA

ELISA plates (Nunc, PolySorp^TM^; Thermo Fisher Scientific, Waltham, MA, USA) were coated with 0.1 mg/mL MWLs in PBS (50 μL/well) and incubated at 4 °C overnight. The wells were washed with water, blocked with 1% (w/v) PEG-4000 for 30 min at RT, and then washed three times with water. The phage diluents in PBS (50 μL/well) were incubated in MWL-coated wells for 2 h at RT, after which the plates were washed four times with PBST and incubated for 1 h at RT with 1:5,000 horseradish peroxidase-conjugated mouse anti-M13 monoclonal antibodies (50 μL/well; GE Healthcare, Buckinghamshire, UK). After washing the plates three times with PBST, *o*-phenylenediamine dihydrochloride (Sigma) was added as a substrate, and the absorbance at 490 nm was measured using a microplate reader Infinite M200 (TECAN, Männedorf, Switzerland).

### Peptide synthesis and concentration determination

The peptides selected for use in binding analysis were synthesised using solid-phase peptide synthesis (Life Technologies Japan, Tokyo, Japan). The concentrations of peptides were determined by measuring UV absorption^40^.

### SPR analysis

The affinity of peptides for MWLs was determined based on SPR measurements performed using a compact SPR sensor SPR02 (OPTOQUEST, Saitama, Japan). After the sensor chips were cleaned using piranha solution (7:3 H_2_SO_4_:30% H_2_O_2_), lignins were immobilised on the chips outside the SPR system. MWLs were deposited onto the bare-gold surface or self-assembled monolayer (SAM)-modified gold surface using a physical immobilisation technique as per the SPR instruction manual. In the first method, MWLs were immobilised directly on the bare-gold surface using the drop-coat protocol. In the second method, the gold sensor chips were immersed for 18 h in a 1 mM solution of methoxy polyethylene glycol thiol (mPEG-SH; molecular weight [MW]: 5,000; Creative PEGWorks, Chapel Hill, NC, USA), with 50% ethanol as the solvent. A fully covered SAM of mPEG-SH was formed on the gold sensor chip, and this substrate was used as a hydrophobic reference surface. MWLs were immobilised through physical interaction with the sensor chip. MWLs were dissolved in dimethyl sulfoxide at a concentration of 10 mg/mL, and the solution (1 μL) was slowly dripped onto the bare-gold or SAM-modified gold surface. After incubation for 30 min at 25 °C, unbound MWLs were removed by washing with water. This process was repeated three times. The sensor chip bearing the immobilised MWLs was docked into the instrument, and the adsorption of MWLs onto the sensor surface was monitored using SPR02. The procedure of physical deposition-mediated immobilisation used in this study differed from that used by most other investigators. In a previous study, a lignin film was prepared and immobilised using the spin-coating technique^41^. As compared with this previous method, physical immobilisation of lignin offers the advantage of easy fabrication without requiring a spin-coater system. When the peptide solutions were injected over the bare gold-MWL surfaces, the SPR signals obtained were nonspecific and similar to the signals measured for binding on the bare gold used as a reference surface. In order to reduce this nonspecific binding, the bare-gold surfaces were covered with a uniform layer of mPEG-SAM. When we investigated the binding of peptides to the mPEG-modified surface, we detected little nonspecific binding. Therefore, we used the mPEG-SAM surface as a reference and for subsequent lignin-immobilisation experiments. Our results showed that lignin immobilisation was successful; a large difference was detected in the responses between mPEG and mPEG-lignin surfaces.

All measurements were performed in PBS (pH 7.0), which was filtered (0.22 μm) and degassed before use. After the sensor chip was docked, PBS was injected over the chip at a flow rate of 20 μL/min for 20–30 min, and a baseline was established for the sensor surface. For determination of equilibrium binding, analyses were performed at RT and at a flow rate of 20 μL/min. Solutions containing synthetic peptides suspended in PBS were used as the samples. The synthetic peptide solutions were injected for 240 s, after which the unbound peptides were removed by washing with PBS. The adsorption of the peptides onto the MWL-immobilised sensor surface was then monitored. The binding of peptides to the sensor surface was marked by corresponding SPR angle changes. The sensor-chip surfaces were regenerated using 10 mM glycine-HCl (pH 2.0).

Binding affinities were calculated from a Scatchard analysis^28^ of equilibrium binding, based on the assumption of a 1:1 binding model, using the equation


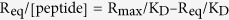


where [peptide], *R*_max_, and *K*_D_ are the peptide concentration, maximum resonance, and dissociation constant, respectively. The resonances in the association phase at 225 s were used as the equilibrium resonance units (*R*_eq_). The *R*_eq_ values were plotted in the form of *R*_eq_/[peptide] versus *R*_eq_, and *K*_D_ and *R*_max_ were calculated from the slope and the intercept of the linear part of the plot, respectively. The *K*_D_ values were determined over the peptide concentration range of 12.5–300 μM. For C401 and E405, analysis was performed over the concentration range of 50–200 μM because reliable signals were not detected below 50 μM.

### CD spectroscopy

CD spectroscopy was performed on a Jasco J-820 spectropolarimeter using a 1-mm cuvette. Spectra were collected every 0.5 nm from 400 to 180 nm. The peptide solution was prepared using ultrapure water. The CD spectra are reported as mean residue ellipticity ([Φ]) in degrees per square centimetre per decimole.

### Molecular modelling

Molecular modelling of the lignin-binding peptides was performed using the Spartan’10 and MS-Forcite programs bundled in Materials Studio software package. The initial structure of the peptide C416 was built using Spartan, where the main chains were set to form a typical α-helix structure. Simple geometry optimisation and conformation searches were performed using the MS-Conformers program with Monte Carlo-based random sampling under a universal force field. After optimisation, reasonable helical structures were extracted for the drawing shown in Fig. 8.

## Additional Information

**How to cite this article**: Yamaguchi, A. *et al.* Discovery of 12-mer peptides that bind to wood lignin. *Sci. Rep.* **6**, 21833; doi: 10.1038/srep21833 (2016).

## Figures and Tables

**Table 1:** Phage yields from each biopanning round of selection against MWL.

	Input phage (pfu/mL)	Output phage (pfu/mL)	Yield^*^ (%)
**For CMWL**			
Round 1	1.5 × 10^12^	8.8 × 10^7^	0.0058
Round 2	1.4 × 10^12^	3.1 × 10^6^	0.0002
Round 3	1.0 × 10^11^	1.2 × 10^6^	0.0012
Round 4	1.8 × 10^10^	6.0 × 10^5^	0.0033
**For EMWL**			
Round 1	1.5 × 10^12^	6.6 × 10^7^	0.0044
Round 2	1.3 × 10^12^	2.9 × 10^8^	0.0220
Round 3	1.0 × 10^11^	1.6 × 10^6^	0.0016
Round 4	1.8 × 10^10^	6.6 × 10^6^	0.0037

*Percent yields of output phage relative to input phage are calculated.

**Table 2:** Amino acid sequences of the phage clones selected for binding to lignin.

Biopanning for CMWL				Biopanning for EMWL			
Clone	Sequence	Frequency	p*I*^a^	Clone	Sequence	Frequency	p*I*^a^
C402	QHRFANHLIFKT	1/34	11.00	E312	MNRHSSLPLKPW	1/36	11.00
C312	KIWIPPKPMSPW	1/34	10.00	E401	VQHNRKYSVVIR	1/36	11.00
C414	WHKPWMFGKLTQ	1/34	10.00	E402	WTPNKLKTMQVK	1/36	10.30
C416	HFPSPIFQRHSH	6/34	9.77	E318	AHNVMVATKIPK	1/36	10.00
C315	LAPRHTHSIHPS	1/34	9.77	E406	AITHGAKMPAKI	1/36	10.00
C415	HRQNVSQPVNPQ	1/34	9.76	E405	VQHNTKYSVVIR	4/36	9.99
C418	HPSSPTPSPWRF	1/34	9.76	E412	LPTKTLYPHVRM	1/36	9.99
C413	LSATSRLQFPSI	1/34	9.75	E417	STVKYHNHNRNF	1/36	9.99
C306	MHTQRTPWIFSL	1/34	9.52	E411	VPHMAPHRIAAQ	1/36	9.73
C309	SMGPTRTPPPNT	1/34	9.47	E414	FATNHRTTHERI	1/36	9.61
C411	HYKYYPTASVMK	1/34	9.40	E416	DHAARNWVERQR	1/36	9.52
C310	HSAKLWLIPSMS	1/34	8.76	E308	LTLNKHPNSHHI	1/36	8.77
C313	GLKVWTVQPPHV	1/34	8.76	E302	KLSNFHPQGSMM	1/36	8.76
C417	HFNRDWQKIHGP	1/34	8.76	E408	KGPHFPSPHVAL	2/36	8.76
C405	HPIWYPTNINRQ	1/34	8.75	E404	VQHNTKYSDVIR	1/36	8.57
C412	KVWTIDTAHTRA	1/34	8.75	E410	VQHHHSYKGVTY	1/36	8.49
C303	YTQVPTKMQLGG	1/34	8.59	E407	YHPNGMNPYTKA (=C401)	2/36	8.50
C401	YHPNGMNPYTKA (=E407)	4/34	8.50	E304	THLGLYQRNTMN	1/36	8.44
C316	QMKCCIATYNPP	1/34	8.05	E403	SHEPVLMIQKFK	2/36	8.34
C314	SYGLKPFMPWYS	1/34	8.22	E311	SKHNYPSQGPVF	1/36	8.33
C403	GAFHVWQPTVTM	1/34	6.74	E313	QKTNHHAHIWDG	1/36	7.02
C404	HQSKISIAVDQP	1/34	6.74	E303	HHGWVSPQYGVA	1/36	6.92
C308	VNNHWDNSHPNT	1/34	5.97	E307	LPKHNEHYFTMP	1/36	6.92
C406	SSWWGEGMNKSY	1/34	5.72	E315	LVNYQSELHQTR	1/36	6.75
C307	QMSITLQNSYLI	1/34	5.52	E314	DMKWTLKEWMTH	1/36	6.75
C317	SLDAWEVERRST	1/34	4.68	E310	GQHNTNGNQTIT	1/36	6.74
				E316	SKMAHMERSWEV	2/36	6.48
				E306	VSLNTDYWNRNY	1/36	5.81
				E301	QEQDWTNSQRIN	1/36	4.37

Peptide sequences are sorted according to p*I*. The “HFPSP” sequences are underlined.

^a^Isoelectric point (p*I*) values of peptide sequences were calculated using ExPasy.

**Table 3:** Frequency of amino acid residues in the peptides selected through biopanning.

Amino acid	Frequency (%)	
	C MWL	E MWL
G	3.43	3.24
A	3.68	4.40
V	3.19	8.56
L	3.43	4.40
I	5.39	3.94
M	3.92	4.63
F	5.15	2.08
W	4.17	2.55
P	13.24	6.48
S	9.80	6.25
T	6.86	7.18
N	5.39	8.10
Q	5.88	5.56
C	0.49	0
D	1.23	1.39
E	0.74	2.78
K	4.90	7.87
H	10.29	10.65
R	4.41	5.09
Y	4.41	4.86

Frequencies indicate the fraction of the amino acid in all isolated clones from the third and fourth rounds of biopanning.

**Table 4:** Dissociation constants measured for the interaction of synthetic peptides with lignin.

Synthetic peptide	*K*_D_ [10^−5 ^M]	
	CMWL	EMWL
C416	10.3	7.0
C401	69.3	72.5
E401	15.5	7.4
E405	22.7	22.7

**Table 5:** Single-residue-substituted mutants of peptide C416.

Clone	Sequence^a^	p*I*^b^
C416	HFPSPIFQRHSH	9.77
C416-1a	HFPSGIFQRHSH	9.77
C416-1b	HFGSPIFQRHSH	9.77
C416-2a	HFPIPIFQRHSH	9.77
C416-2b	HFPSPIFQIHSH	7.02
C416-2c	HFPSPIFIRHSH	9.77
C416-2d	HFPSPIFQRHIH	9.77
C416-3a	IFPSPIFQRHSH	9.76
C416-3b	HIPSPIFQRHSH	9.77
C416-3c	HFPSPIFQRISH	9.76
C416-3d	HFPSPIFQRHSI	9.76
C416-3e	HFPSPIIQRHSH	9.77

^a^Substitutions of amino acids are underlined.

^b^Isoelectric point (p*I*) values of peptide sequences were calculated using ExPasy.
